# Metformin partially reverses the inhibitory effect of co-culture with ER^-^/PR^-^/HER2^+^ breast cancer cells on biomarkers of monocyte antitumor activity

**DOI:** 10.1371/journal.pone.0240982

**Published:** 2020-10-27

**Authors:** Zoheir Dahmani, Lynda Addou-Klouche, Florence Gizard, Sara Dahou, Aida Messaoud, Nihel Chahinez Djebri, Mahmoud Idris Benaissti, Meriem Mostefaoui, Hadjer Terbeche, Wafa Nouari, Marwa Miliani, Gérard Lefranc, Anne Fernandez, Ned J. Lamb, Mourad Aribi

**Affiliations:** 1 Laboratory of Applied Molecular Biology and Immunology, W0414100, University of Tlemcen, Tlemcen, Algeria; 2 Cell Biology Unit, IGH CNRS, Université de Montpellier, (UMR 9002), Montpellier, France; 3 IGH, UMR 9002 CNRS-Université de Montpellier, Montpellier, France; Duke University School of Medicine, UNITED STATES

## Abstract

**Background:**

Immune activities of monocytes (MOs) can be altered within the microenvironment of solid malignancies, including breast cancer. Metformin (1,1-dimethylbiguanide hydrochloride, MET), has been shown to decrease tumor cell proliferation, but its effects have yet to be explored with respect to MOs (monocytes) activity during their crosstalk with breast cancer cells. Here, we investigated the effects of MET on overall phenotypic functional activities, including cellular immunometabolism and protective redox signaling based-biomarkers, intracellular free calcium ions (_if_Ca^2+^), phagocytosis and co-operative cytokines (IFN-γ and IL-10) of autologous MOs before and during their interplay with primary ER^-^/PR^-^/HER2^+^ breast cancer cells.

**Methods:**

Human primary breast cancer cells were either cultured alone or co-cultured with autologous MOs before treatment with MET.

**Results:**

MET downregulated breast cancer cell proliferation and phagocytosis, while having no significant effect on the ratio of phosphorylated Akt (p-Akt) to total Akt. Additionally, we observed that, in the absence of MET treatment, the levels of lactate dehydrogenase (LDH)-based cytotoxicity, catalase, _if_Ca^2+^, IL-10 and arginase activity were significantly reduced in co-cultures compared to levels in MOs cultured alone whereas levels of inducible nitric oxide synthase (iNOS) activity were significantly increased. In contrast, MET treatment reduced the effects measured in co-culture on the levels of LDH-based cytotoxicity, arginase activity, catalase, _if_Ca^2+^, and IFN-γ. MET also induced upregulation of both iNOS and arginase in MO cells, although the increase did not reach significant difference for iNOS activity. Moreover, MET induced a robust increase of superoxide dismutase (SOD) activity in MOs, but not in MOs co-cultured with breast cancer cells. Furthermore, MET markedly upregulated the levels of IFN-γ production and downregulated those of IL-10 in isolated MOs, while inducing a slight opposing up-regulation of IL-10 production in co-cultures.

**Conclusions:**

Our results show that the biomarkers of phenotypic functional activities of MOs are modified after co-culturing with primary human breast cancer cells. Treatment of co-cultures with MET resulted in increased release of antitumor cytokine IFN-γ and _if_Ca^2+^, and increased cell necrosis during breast cancer cells-MOs crosstalk.

## Introduction

Breast cancer is the most commonly diagnosed cancer and a leading cause of mortality worldwide [1]. Compared to other types of cancer that are considered as more responsive to immunotherapy, breast cancer has not been traditionally considered as an immunogenic malignancy [2]. However, recent research has shown the relationship between immune intra-tumoral responses and breast cancer development [3]. Additionally, studies reported that infiltration of immune cells within the tumor microenvironment and the presence of immunity-related gene signatures contribute to breast cancer prognosis [4,5].

The microenvironment surrounding breast cancer cells plays an important role in modulating cancer growth and progression [3]. It contains several types of inflammatory cells including MOs and macrophages. MO cells represent a heterogeneous population derived from myeloid lineages [6] that are recruited from the bloodstream to the tumor site through the paracrine action of cytokines and chemokines released by breast cancer cells [7]. Previous reports suggested that infiltration of MOs into the breast tumor microenvironments, in response to paracrine stimulation, correlates with poor prognosis and promotion of tumor growth, invasion and metastasis [8,9].

In light of their functional phenotypic plasticity, MOs can be targeted by several therapeutic molecules that switch them towards proinflammatory/anti-tumoral killer cells [10,11], which are mainly implicated in inflammatory response, thereby having reduced phagocytic capacity [12]. In context of cancer, these cells exert their inhibitory effects by enhanced production of proinflammatory cytokines, like IFN-γ, secretion of tumoricidal mediators, reactive oxygen (ROS) and nitrogen species (RNS), including the production of nitric oxide (NO) as product of the NOS activation [13].

It is well known that insulin is an important growth factor, which plays a critical role in regulation of cell proliferation. As such, enhancing insulin sensitivity can lead to tumor growth inhibition and cell cycle arrest. Indeed, metformin (1,1-dimethylbiguanide hydrochloride, MET), an antidiabetic drug prescribed for patients with type 2 diabetes [14,15], has been reported to have a marked effect on insulin sensitivity through inhibition of the signaling pathway implicating phosphoinositol-3-kinase (PI3K) and Akt (also referred to as protein kinase B, PKB) consequently leading to decreased tumor cell proliferation [16,17]. The effects of MET on breast cancer cells has also been associated with the inhibition of pro-tumoral M2-like macrophage polarization [18]. In this context, we investigated for the first time the effects of MET on the overall phenotypic functional activities, including immunometabolic (arginase activity, iNOS activity and LDH release) [19] and protective redox based-biomarkers (catalase and SOD activities) [20], _if_Ca^2+^, phagocytosis, and co-operative cytokines (IFN-γ and IL-10) [21] of autologous MOs before and during their crosstalk with breast cancer cells (ER^-^/PR^-^/HER2^+^).

## Materials and methods

### Materials

Unless specified, all materials including (MET), were obtained from Sigma-Aldrich (Sigma Chemical Co., St. Louis, USA).

#### 1. Study design

**Fig 1:**
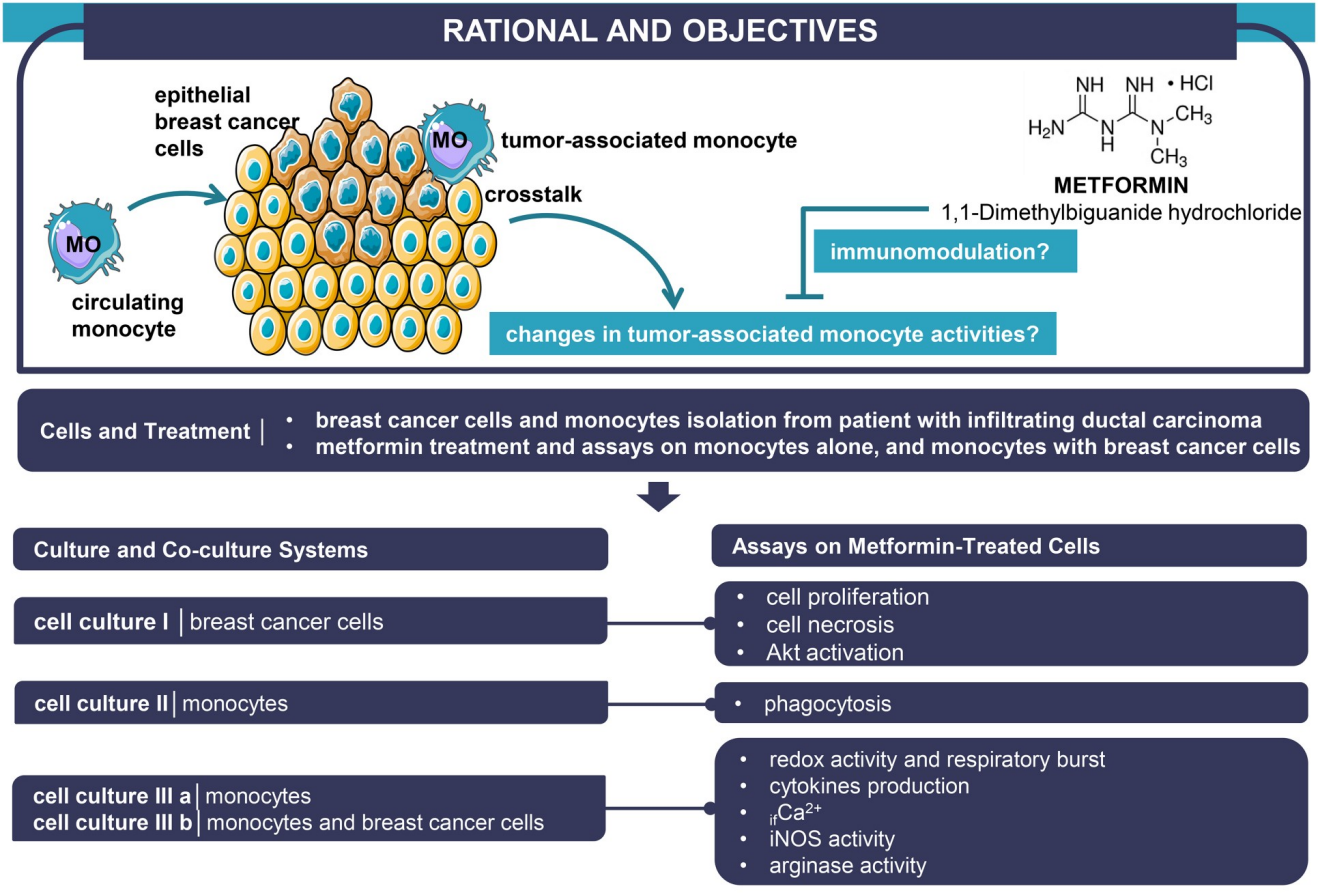
Summary of experimental design. Akt: protein kinase B (PKB), _if_Ca^2+^: intracellular free calcium ions, iNOS: nitric oxide synthase, MO: monocyte.

Tumor epithelial cells were isolated from breast cancer tissue specimens, and co-cultured with autologous MOs, isolated from peripheral blood mononuclear cells (PBMCs). First, tumor cells were cultured alone to check the MET effects on both proliferation and viability using BrdU (Bromodeoxyuridine [5-bromo-2’-deoxyuridine]), and Trypan Blue Exclusion Test [TBET], respectively, and on p-Akt-to-Akt ratios. Similarly, MOs were cultured alone for phagocytosis capacity assays. LDH-based cytotoxicity, respiratory burst and redox activity (nitric oxide [NO], catalase, superoxide dismutase [SOD]), release of co-operative cytokines (‘antitumor cytokine IFN-γ’, and ‘immunosuppressive/regulatory cytokine IL-10’), inducible nitric oxide synthase iNOS-associated proinflammatory MOs and arginase activities-associated anti-inflammatory MOs, and intracellular free calcium ions (_if_Ca^2+^) were measured in MOs cultured alone and co-cultured with breast cancer cells. All experiments were repeated four times. The experimental approach is outlined in the graphical abstract, Fig 1. The purity of MOs was verified by direct immunofluorescence (S1 Fig).

#### 2. Specimen samples

Primary tumor tissue specimens and autologous peripheral blood samples were collected thanks to three patient volunteers, admitted to the Surgery Department of Tlemcen Medical Centre University (Algeria), who have been newly diagnosed for human epidermal growth factor receptor 2-Positive/estrogen receptor-Negative/progesterone receptor-Negative (ER^-^/PR^-^/HER2^+^) breast cancer (age group 50–60 years), and who have not yet begun treatment, after obtaining informed and written consent from each to participate to the current study. Peripheral blood was collected in heparinized *Vacutainer* tubes (BD, Belliver Industrial Estate, UK). The homogeneity of sample biopsies and absence of intertumor variations for each tumor intended for experimental analyses were checked by macroscopical and thorough anatomopathological examinations, and based on cellular morphology and immunohistochemical analyses. All tumor samples are grade 2 nonspecific invasive mammary carcinoma classified as pT2N0. The current study was approval by local Ethics Committee of Tlemcen University, in accordance with the Declaration of Helsinki.

#### 3. Isolation of mammary adherent tumor epithelial cells

After removal of the healthy tissue surrounding the tumor tissue, tumor mammary epithelial cells presenting as infiltrating ductal carcinomas were isolated from primary cancer specimens by enzymatic digestion and differential centrifugation according to Feller *et al*., and Speirs *et al*. [22,23], with some modifications. Briefly, the tumor tissue specimens were washed extensively with 1x phosphate saline buffer (PBS), placed in sterile Petri dishes and cut into small 2 mm pieces with a sterile scalpel. The minced tissue was incubated in 0.1% collagenase solution at 37°C for 12–20 h. Following digestion, cell mixtures were centrifuged at 40 x *g* for 1 min and the supernatant transferred to new tubes that were then centrifuged at 100 x *g* for 2 min to obtain a pellet representing tumor epithelial cells.

#### 4. Cell culture

Epithelial cancer cells were washed with RPMI-1640 medium, supplemented with 10% fetal calf serum (FCS) and 50 μg/mL gentamicin, by centrifugation at 40 x *g* for 5 min. The cell pellet was resuspended with 10 mL of RPMI-1640 supplemented with 10% FCS and 50 μg/mL gentamicin and then subdivided into two culture flasks and incubated in a humidified atmosphere at 37°C and 5% CO_2_. Fibroblast contamination was removed from epithelial cancer cells by differential trypsinization [24]. The culture medium was changed every 2–5 days [25]. Cells were passaged with 0.25% trypsin-EDTA (ethylenediamine tetraacetic acid) when they reached ~80% confluence [26].

#### 5. Peripheral blood mononuclear cells isolation

Blood samples were diluted 1:1 with PBS and layered on Histopaque-1077 (Sigma-Aldrich, St. Louis MO, USA) and centrifuged at 400 x *g* for 30 min. The interface band containing PBMCs was carefully harvested washed twice with PBS. Cell pellets were suspended in 1 mL of RPMI-1640 supplemented with 10% FCS and 50 μg/mL of gentamicin for cell counting. Cell viability was performed by TBET using photonic microscopy (Zeiss, Germany).

#### 6. MOs isolation

MOs were isolated from PBMCs based on differential plastic adherence [27]. Briefly, PBMCs were cultivated in RPMI-1640 supplemented with 10% FCS and 50 μg/mL gentamicin, and seeded at 2 x 10^6^ cell/mL into 24-well plates. Cells were allowed to adhere for 2 h at 37°C before removal of non-adherent cells were and treatment of adherent MOs with MET. Cells were counted microscopically (Zeiss, Germany) using trypan blue staining, and the purity of monocytes was evaluated by fluorescent staining with PhycoErytherin (PE)-anti-human CD14 antibody (BD Biosciences, San Diego, CA, USA) using a Floid Cell Imaging Station (Thermo Fisher Scientific, MA USA) [28,29] and routinely exceeded over 90% purity (S1 Fig).

#### 7. Cell culture and co-culture systems

After cell detachment with trypsin-EDTA [30], breast cancer cells were counted before being cultured alone or co-cultured with an equal number of MOs (2 x 10^5^ cells/mL) at a ratio of 1:1 in RPMI-1640 supplemented with 10% FCS and 50 μg/mL gentamicin.

#### 8. MET treatment

MOs, breast cancer cells or co-cultured MOs with breast cancer cells were treated for 24 h with fresh medium containing or not MET at the dose of 2.5 mM [15].

#### 9. TBET assays

The effect of MET treatment on cancer cell viability was based on TBET. Breast cancer cells (2 x 10^5^ cells per well) were grown overnight in a 24-well plate at 37°C in a humidified atmosphere and 5% CO_2_ for adherence. Thereafter, culture medium was replaced with fresh RPMI-1640 medium containing MET and incubated a further 24 h. Cells were subsequently washed with 1x PBS, trypsinized before determination the number of viable and dead cells with TBET.

#### 10. BrdU assays

Cell proliferation was measured by BrdU incorporation using a BrdU Cell Proliferation ELISA according to the manufacturer’s instructions (ab126556-BrdU Cell Proliferation kit, Abcam, Germany). Briefly, breast cancer cells (2 x 10^5^ cells/mL) were treated with MET in 96-well microplates for 24 h at 37°C in a humidified atmosphere and 5% CO_2_. Thereafter, 20 μL of the diluted 1x BrdU was added to each well and cells were incubated overnight. Cells were then fixed and BrdU incorporation detected using anti-BrdU monoclonal Detector Antibody for 1 h at room temperature before incubation with peroxidase goat anti-mouse IgG conjugate as secondary antibody. Color was developed using tetramethylbenzidine (TMB) as a peroxidase substrate and BrdU incorporation measured at 450 nm using an ELISA reader (Biochrom Anthos 2020, Cambridge, UK).

#### 11. Western blotting assays

**Fig 2:**
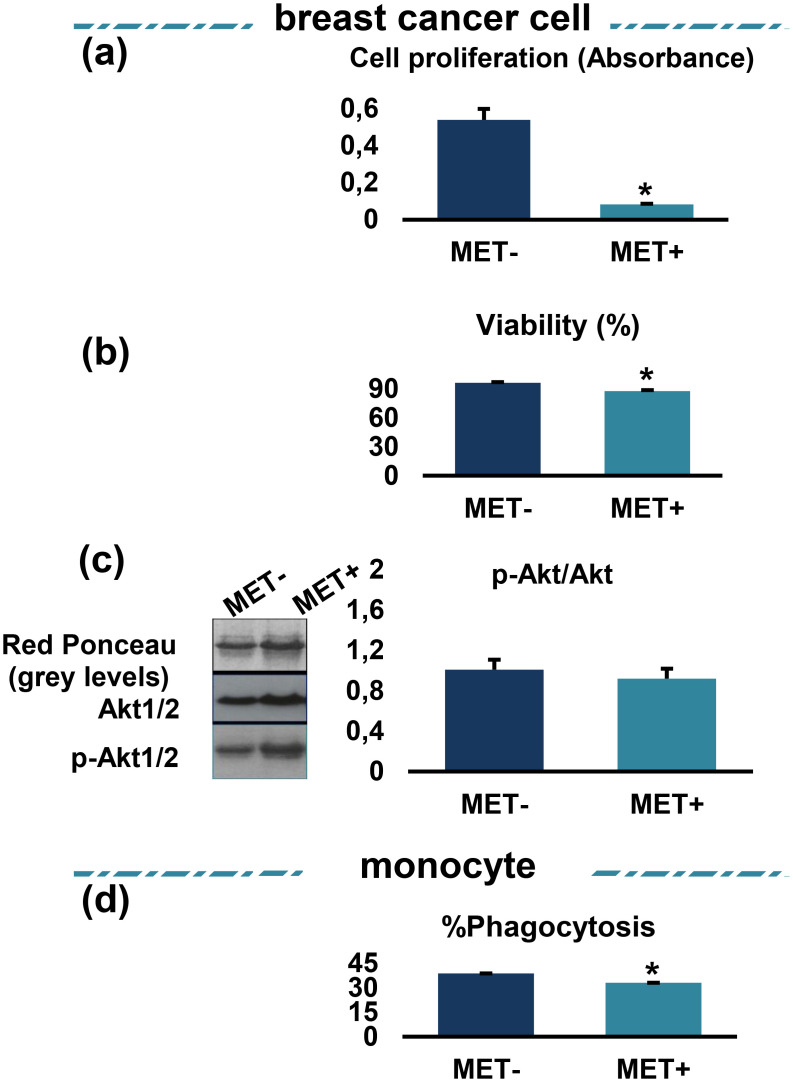
Effect of MET treatment on breast cancer cell proliferation, viability, ratio of phosphorylated Akt-to-total Akt and MOs phagocytosis capacity. In breast cancer cells treated or not with MET, (a) cell proliferation was determined by BrdU assay and (b) viability by TBET assay. (c) phosphoAkt to Akt ratio: levels of major proteins stained by red Ponceau are shown as loading control. Values are represented as a ratio to the protein levels in Ponceau red and with the value for zero MET set as 1. MET: metformin, p-Akt: phosphorylated Akt. (d) MOs were infected with *Staphylococcus aureus* before treatment with MET. The results were expressed as a percentage of phagocytosis. Values are presented as the mean with standard error of mean for four independent experiments carried out on three samples (n = 12 for each group). Asterisks indicate significant differences between treated cells and untreated controls by Mann-Whitney *U* test (*p < 0.05). MET: metformin, MOs: monocytes.

After 24 h incubation of breast cancer cells treated or not with MET, cells were washed with PBS and lysed using Triton X-100. Proteins present in equal amounts of cell lysates were rapidly diluted with SDS-sample buffer (50 mM Tris-HCL pH 6.8, 2 mM DTT, 1.0% SDS), boiled for 5 min. Proteins were separated by 10% sodium dodecyl-sulfate polyacrylamide gel electrophoresis (SDS-PAGE). Protein concentrations were not determined before reduction and denaturation to minimize the chance of protein dephosphorylation. After separation, proteins were transferred to a nitrocellulose membranes and transferred protein were visualized by staining with Ponceau red. Thereafter, membranes were blocked with 5% nonfat milk or 5% bovine serum albumin (BSA) for 45 min at room temperature and incubated overnight at 4°C with primary antibodies against p-Akt (Ser473) (1/1000), Akt-1 (2H10) (1/1000), Akt-2 (5B5) (1/1000) Cell Signaling Technology (Denvers, MA, USA). Horseradish peroxidase-conjugated (HRP) anti-mouse IgG and anti-rabbit IgG were used as secondary antibodies for 1 h at room temperature. Blotted membranes were detected with enhanced chemiluminescence reagent (Amersham Pico) using X-ray film. Quantitative analysis of the signals from scanned films was performed using Imgcalc2, a unix software developed in house (IGH, Montpellier) for quantifying pixels on numerical images. The results in Fig 2 are represented as a ratio to the signal in Ponceau Red staining to correct for differences in total protein loading with the levels for MET at dose 0 set as 1.

#### 12. Phagocytosis assay

Assay for phagocytosis capacity was performed as described [31–33]. Briefly, a total of 2 x 10^5^ MOs were infected with *Staphylococcus aureus* at multiplicity of infection (MOI) of 50 in a 24-well plate and incubated with MET for 1 h at 37°C in a 5% CO_2_ incubator. The number of viable bacteria was determined by serial dilution and colony forming unit (CFU) counts on Chapman medium. The percentage of phagocytosis was calculated as follows:

$$Phagocytosis\text{\%}=Mto-100\times \frac{\left(\frac{NEC}{NC1/NC0}\right)}{Mt0}$$

M_t0_ is the number of bacteria in the assay sample mixture at t_0_. NEC, number of extracellular bacteria in assay mixtures sample at t_1_. NC_0_ and NC_1_ correspond to control sample at t_0_ and t_1_.

#### 13. Arginase activity assay

The enzymatic activity of arginase (EC 3.5.3.1) was evaluated in cell lysates based on determination of urea levels following the L-arginine hydrolysis as described in detail [34]. The arginase activity was expressed as mU urea/mg protein/1 h.

#### 14. iNOS activity assay

Measurement of iNOS activity was based on the determination of NO generation levels. The accumulation of NO in cell-free culture supernatants were evaluated by nitrite (NO_2_) measurement, as stable and final end-product of NO, using a sensitive colorimetric Griess reaction as described in detail [34,35]. Absorbance was measured at 540 nm using a Biochrom Anthos 2020 ELISA plate reader. NO production levels were calculated by comparison with a sodium nitrite (NaNO_2_) curve standard [36]. iNOS activity was obtained by normalizing each NO concentration to milligrams of protein and expressed as picomoles/mg protein/30 min.

#### 15. LDH-based cytotoxicity assays

LDH-based cytotoxicity levels were determined by evaluation of LDH release into the cell culture supernatants using Lactate Dehydrogenase Activity Assay kit (MAK066, Sigma-Aldrich). Briefly, 50 μL of supernatant and 50 μL of the Master Reaction Mix were mixed and added to each well of a 96-well plate. Absorbance was measured at 450 mn after 30 min incubation at 37°C in accordance with the manufacturer’s instructions.

#### 16. _if_Ca^2+^ assay

The concentrations of _if_Ca^2+^ were measured biochemically based on the ortho-cresolphthalein complexone (oCPC) method as described elsewhere [37].

#### 17. Cytokine assays

Concentration of IFN-γ and IL-10 in cell culture media of MOs or co-culture system supernatants were measured by sandwich enzyme-linked immunosorbent assays (ELISA), using respective commercial kits (BD Biosciences), according to the manufacturer’s instructions. Optical densities (OD) were measured at 450 nm using appropriate standard curves for each cytokine.

#### 18. Protective redox activity assays

Redox activity was evaluated by determination of the levels of catalase and SOD activities.

*18*.*1*. *Catalase activity assay*. The enzymatic activity of catalase was spectrophotometrically determined in cell lysates by measurement of hydrogen peroxide (H_2_O_2_) decomposition [38]. 10 μL volumes of cell lysates were added to a reaction mixture of H_2_O_2_ in 0.9% (v/v) aqueous saline before incubation for 5 min. The reactions were stopped by the addition of Titanyl sulfate (TiOSO_4_), and the absorbance measured at 410 nm.

*18*.*2*. *SOD activity assay*. SOD activity in cell lysates was determined spectrophotometrically by measuring production of a water-soluble formazan dye resulting from the reduction of Dojindo’s highly water-soluble tetrazolium salt WST-1 ((2-4-Iodophenyl)-3-(4-nitrophenyl)-5-(2,4-disulfophenyl)-2H-tetrazolium, monosodium salt), using a SOD Assay Kit-WST (19160, Sigma Aldrich). Twenty μL of the enzyme working solution were added to a mixture containing 20 μL of cell lysate and 200 μL of WST Working Solution. The microplate was incubated at 37°C for 20 min, and the absorbance read at 440 nm. The SOD activity (percentage inhibition of WST-1 inhibition) was calculated as follows:

$$SODactivity(inhibitionrate\text{\%})=\frac{\left(Ablank1–Ablank3)–(Asample–Ablank2\right)}{\left(Ablank1-Ablank3\right)}x100$$

#### 19. Statistical analysis

Data are presented as the mean with standard errors of means (SEM). Statistical analyses were performed using non-parametric Mann-Whitney *U* or Kruskal-Wallis one-way analysis of variance (ANOVA) test with pairwise comparisons using the Dunn–Bonferroni approach after checking the distribution of data. Statistics were carried out using IBM SPSS Statistics version 20. *P*-values less than 0.05 were considered significant.

## Results

### 1. MET effects on breast cancer cells and on monocytes cultured alone

#### 1.1. MET downregulates breast cancer cell viability and proliferation, while has no effect on the ratio of phosphorylated Akt1/2 *versus* total Akt1/2

As shown in Fig 2a and 2b, MET treatment significantly downregulated breast cancer cell proliferation and viability levels (for both comparisons, *p* < 0.05). Fig 2c shows the raw data of the actual protein levels (upper panels), total Akt1/2 levels (middle panels) and levels of phospho-(activated) Akt1/2 (lower panels). The histogram shows the relative expression levels of activated Akt1/2-to-total Akt1/2 ratio after normalization to the total protein loaded. As observed in Fig 2c, MET treatment did not show a significant difference in either Akt levels and ratio of activated Akt *versus* total Akt when comparing with MET-untreated cells (*p* > 0.05). So the actions of MET on Akt1/2 are essentially due to a loss/reduction of Akt1/2 activity since when the ratio of active Akt1/2 (phosphorylated) to total Akt1/2 levels is calculated we observed a reduction of Akt1/2 activity in cancer cells.

#### 1.2. MET downregulates monocyte phagocytosis

As shown in Fig 2d, the phagocytic activity of MOs significantly decreased after MET treatment (*p* < 0.05).

### 2. MET effects on MO cells in monoculture and co-culture systems

#### 2.1. MET might reverse the co-culture effect on LDH-based cytotoxicity levels, but has no cytotoxic effect on MOs cultured alone

**Fig 3:**
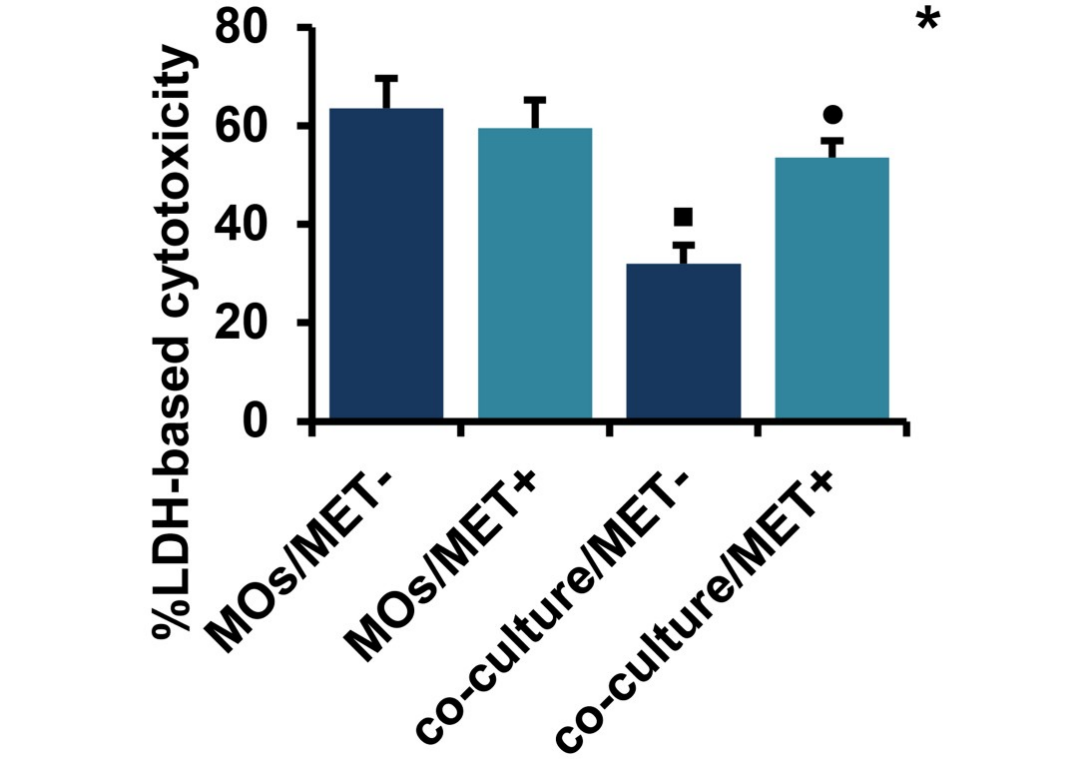
Cytotoxic effect of MET on MOs and co-culture system. Necrosis levels were measured spectrophotometrically through the evaluation of LDH release. Values are presented as the mean with standard error of mean for four independent experiments carried out on three samples (n = 12 for each group). MET: metformin, LDH: lactate dehydrogenase, MOs: monocytes, MOs/MET-: MET-untreated MOs, MOs/MET+: MET-treated MOs, co-culture/MET-: MET-untreated co-culture system, co-culture/MET+: MET-treated co-culture system. Black dots indicate significant differences when comparing each treated group with untreated controls (0 mM MET) using Mann-Whitney *U* test (•*p* < 0.05). Black boxes indicate significant differences highlighted between MET-untreated MOs and MET-untreated co-culture system using Mann-Whitney *U* test (▪*p* < 0.05). Asterisks indicate significant differences highlighted between all groups by Kruskal-Wallis test with pairwise Dunn-Bonferroni adjustment (*p < 0.05).

As shown in Fig 3, MET treatment induced no necrosis/LDH-based cytotoxicity effects on MO cells (*p* > 0.05). Additionally, MET might reverse the co-culture effect on LDH-based cytotoxicity levels. Conversely, the level of LDH-based cytotoxicity was significantly downregulated in MET-untreated co-cultures of MOs with breast cancer cells when compared to MET-untreated MOs cultured alone (*p* < 0.05).

#### 2.2. MET ameliorates simultaneously iNOS and arginase activities in co-cultures of MOs with breast cancer cells

**Fig 4:**
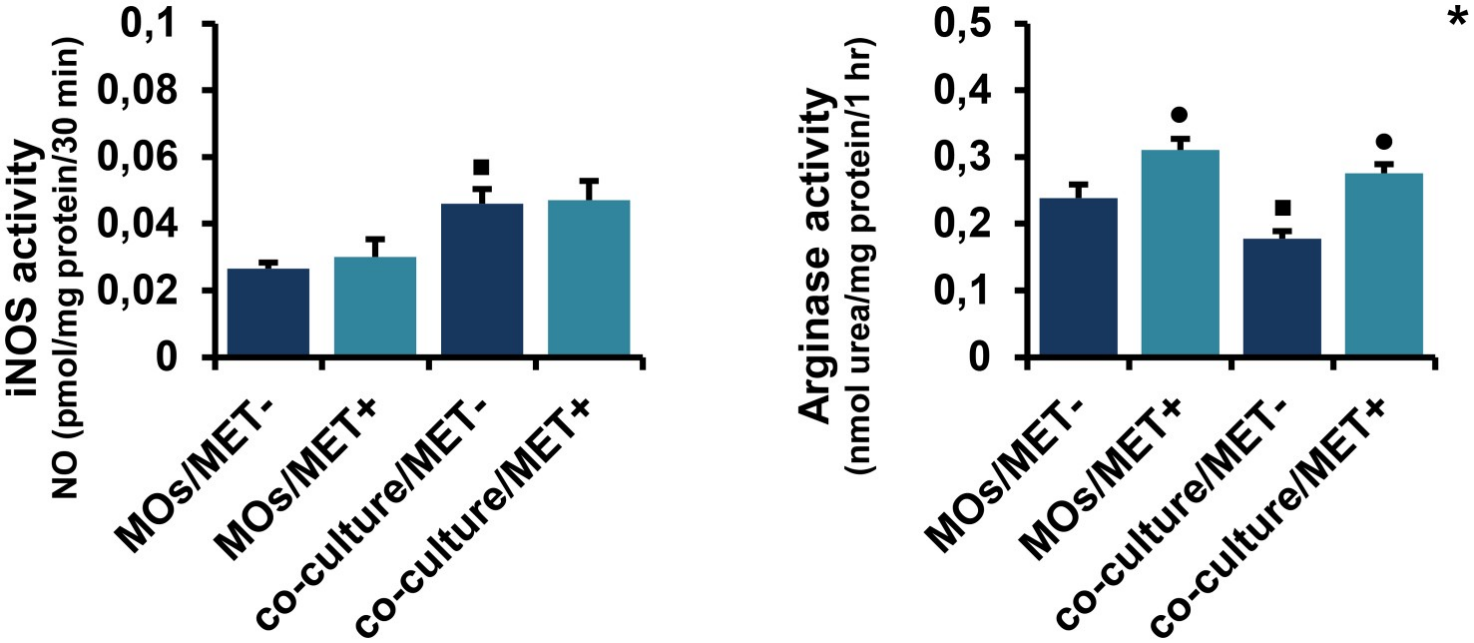
Effect of MET on iNOS and arginase activities in MOs and co-culture system. NO levels were measured by Griess colorimetric reaction and iNOS activity was obtained by normalizing each NO to protein concentrations and time. The enzymatic activity of arginase was evaluated in cell lysates by the spectrophotometric measurement of urea concentration. Values are presented as the mean with standard error of mean for four independent experiments carried out on three samples (n = 12 for each group). MET: metformin, MOs: monocytes, NO: nitric oxide, iNOS: inducible nitric oxide synthase, MOs/MET-: MET-untreated MOs, MOs/MET+: MET-treated MOs, co-culture/MET-: MET-untreated co-culture system, co-culture/MET+: MET-treated co-culture system. Black dots indicate significant differences when comparing each treated group with untreated controls (0 mM MET) using Mann-Whitney *U* test (•*p* < 0.05). Black boxes indicate significant differences highlighted between MET-untreated MOs and MET-untreated co-culture system using Mann-Whitney *U* test (▪*p* < 0.05). Asterisks indicate significant differences highlighted between all groups by Kruskal-Wallis test with pairwise Dunn-Bonferroni adjustment (*p < 0.05).

As depicted in Fig 4, MET induced an increase in iNOS activity in co-culture systems, as compared to MET-treated or MET-untreated MOs cultured alone; while the difference was not significant for the comparison with MET-treated MOs (respectively, *p* > 0.05 and *p* < 0.05). Additionally, iNOS activity was significantly upregulated in MET-untreated MOs co-cultured with breast cancer cells in comparison to MET-untreated MOs cultured alone (*p* < 0.05). Similarly, (Fig 4), MET might improve the co-culture effect on arginase activity and significantly increased the arginase activity of MOs cultured alone (*p* < 0.05). However, arginase activity was significantly downregulated in MET-untreated MOs co-cultured with breast cancer cells compared to MET-untreated MOs cultured alone (*p* < 0.05).

#### 2.3. MET reverses the co-culture effect on catalase activity, but not on SOD activity

**Fig 5:**
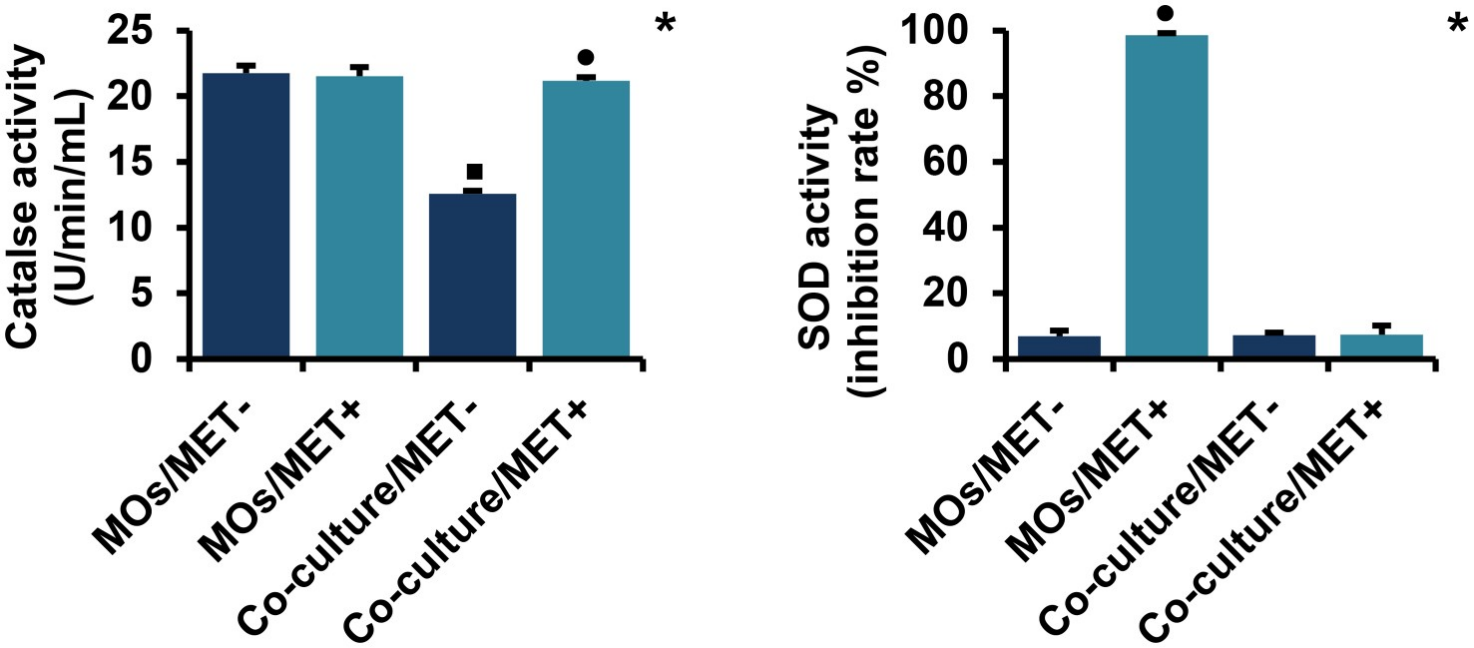
Effect of MET on catalase and SOD activities in MOs and co-culture system. Catalase activity was determined spectrophotometrically by measurement of hydrogen peroxide decomposition. SOD activity was evaluated by spectrophotometric measurement of a water-soluble formazan dye. Values are presented as the mean with standard error of mean for four independent experiments carried out on three samples (n = 12 for each group). MET: metformin, MOs: monocytes, SOD: superoxide dismutase, MOs/MET-: MET-untreated MOs, MOs/MET+: MET-treated MOs, co-culture/MET-: MET-untreated co-culture system, co-culture/MET+: MET-treated co-culture system. Black dots indicate significant differences when comparing each treated group with untreated controls (0 mM MET) using Mann-Whitney *U* test (•*p* < 0.05). Black boxes indicate significant differences highlighted between MET-untreated MOs and MET-untreated co-culture system using Mann-Whitney *U* test (▪*p* < 0.05). Asterisks indicate significant differences highlighted between all groups by Kruskal-Wallis test with pairwise Dunn-Bonferroni adjustment (*p < 0.05).

As demonstrated in Fig 5, MET had no significant effect on catalase activity in MO cells while might reverse the co-culture effect on catalase activity. Additionally, catalase activity was significantly downregulated in MET-untreated co-cultures of MOs with breast cancer cells than in MET-untreated MOs cultured alone (*p* < 0.05). Moreover, SOD activity was strongly increased in MET-treated compared to MET-untreated MOs (*p* < 0.05). In contrast to MOs cultured alone, MET had no significant effect on SOD activity in co-culture systems.

#### 2.4. The effects of MET on _if_Ca^2+^ differs between MOs cultured alone and MOs co-cultured with breast cancer cells

**Fig 6:**
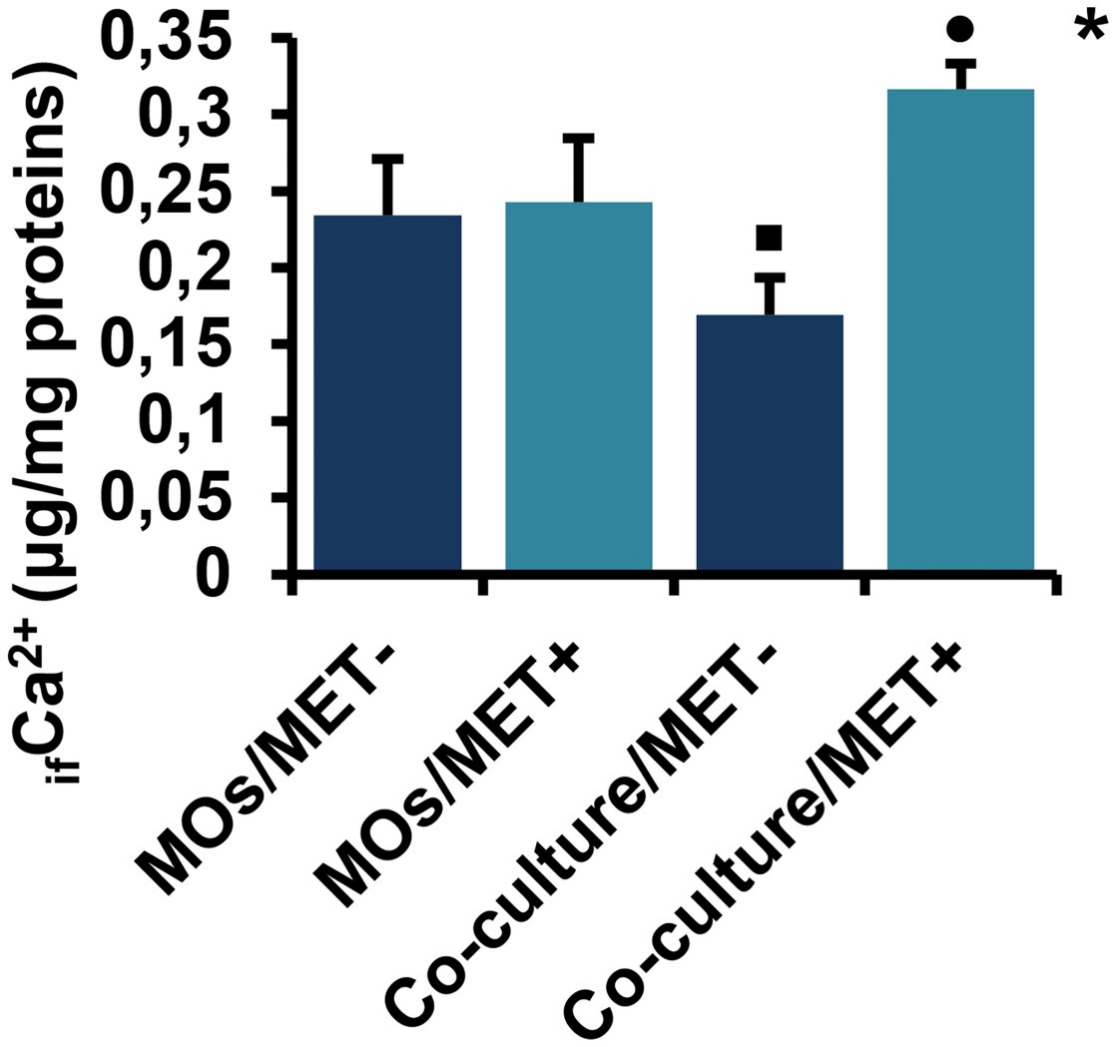
Effect of MET on _if_Ca^2+^ levels in MOs and co-culture system. Values are presented as the mean with standard error of mean for four independent experiments carried out on three samples (n = 12 for each group). MET: metformin, MOs: monocytes, _if_Ca^2+^: intracellular free calcium ions, MOs/MET-: MET-untreated MOs, MOs/MET+: MET-treated MOs, co-culture/MET-: MET-untreated co-culture system, co-culture/MET+: MET-treated co-culture system. Black dots indicate significant differences when comparing each treated group with untreated controls (0 mM MET) using Mann-Whitney *U* test (•*p* < 0.05). Black boxes indicate significant differences highlighted between MET-untreated MOs and MET-untreated co-culture system using Mann-Whitney *U* test (▪*p* < 0.05). Asterisks indicate significant differences highlighted between all groups by Kruskal-Wallis test with pairwise Dunn-Bonferroni adjustment (*p < 0.05).

As shown in Fig 6, MET had no significant effect on _if_Ca^2+^ levels in MO cells while might reverse the co-culture effect on _if_Ca^2+^ levels (*p* < 0.05). Conversely, MET treatment downregulated _if_Ca^2+^ in MET-untreated MOs co-cultivated with breast cancer cells more significantly than in MET-untreated MOs cultured alone (*p* < 0.05).

#### 2.5. MET reverse the effect of co-culture on the production of IFN-γ and IL-10

**Fig 7:**
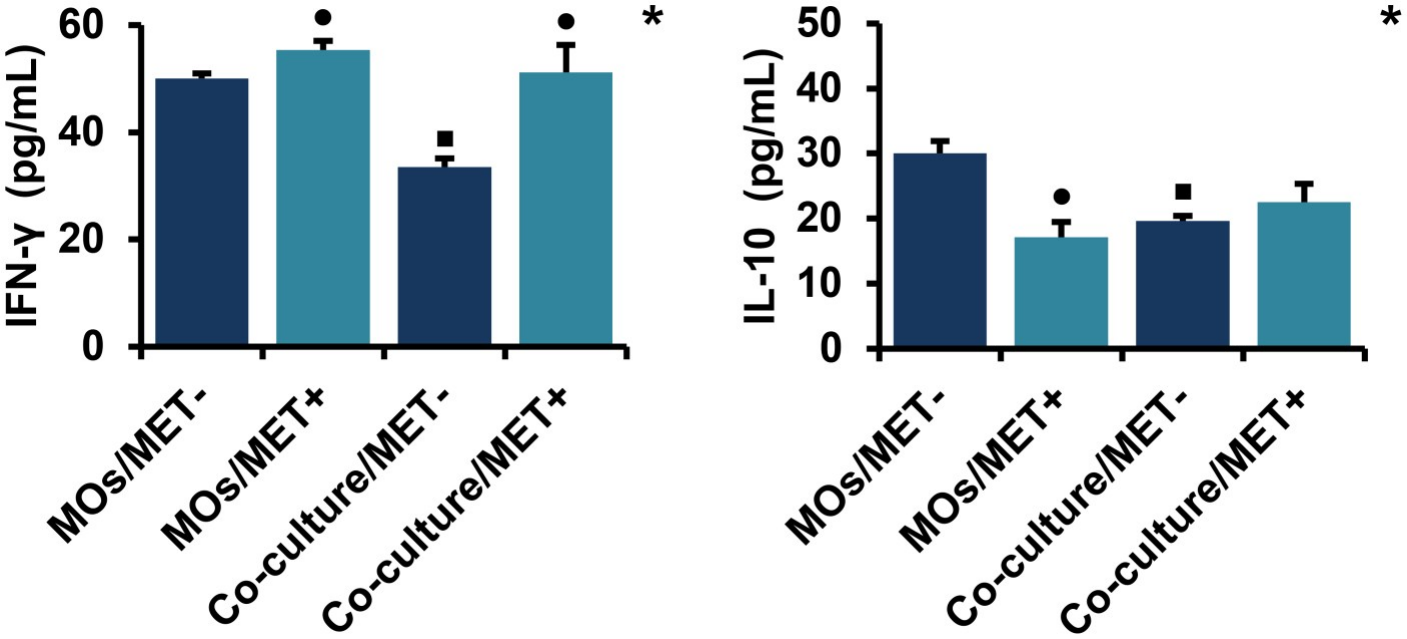
Effect of MET on the production of IL-10 and IFN-γ in MOs and co-culture system. IL-10 and IFN-γ levels were measured using sandwich enzyme-linked immunosorbent assay (ELISA). Values are presented as the mean with standard error of mean for four independent experiments carried out on three samples (n = 12 for each group). MET: metformin, MOs: monocytes, IFN: interferon, IL: interleukin, MOs/MET-: MET-untreated MOs, MOs/MET+: MET-treated MOs, co-culture/MET-: MET-untreated co-culture system, co-culture/MET+: MET-treated co-culture system. Black dots indicate significant differences when comparing each treated group with untreated controls (0 mM MET) using Mann-Whitney *U* test (•*p* < 0.05). Black boxes indicate significant differences highlighted between MET-untreated MOs and MET-untreated co-culture system using Mann-Whitney *U* test (▪*p* < 0.05). Asterisks indicate significant differences highlighted between all groups by Kruskal-Wallis test with pairwise Dunn-Bonferroni adjustment (*p < 0.05).

As shown in Fig 7, MET induced a significant upregulation of IFN-γ levels and a significant downregulation of IL-10 levels in MOs cultured alone (for the two comparisons, *p* < 0.05). Additionally, co-culture induced a significant downregulation of both IFN-γ and IL-10 levels (*p* < 0.05), which are reversed after MET treatment.

## Discussion

MET has recently received increasing attention as a potential therapeutic treatment against cancer [39]. Here we have examined the effects of MET in a novel co-culture system comprising primary MOs and ER^-^/PR^-^/HER2^+^ breast cancer cells.

Breast tumors characterized by overexpression of HER2 have been correlated with increased tumor aggressiveness, invasiveness and poorer prognosis [40]. Measuring several different biomarkers of phenotypic functional activities of MOs before and during their interplay with primary ER^-^/PR^-^/HER2^+^ breast cancer cells, we confirm scientific relevance of the co-culture system over the use of isolated cell types for analyzing the reversing effects of MET in a tumor-like microenvironment, where cancer cells usually alter immune cell functions, especially affecting their cell metabolism and ability for the production of antitumor cytokines, like co-operative cytokines IFN-γ and IL-10.

The dose of MET used in these experiments (2.5 mM) is relatively higher than those used clinically for the treatment of type 2 diabetes (ca 0.5 mM). However, it is important to note that *in vitro* cultured cells are maintained under less physiological conditions. In particular, cultured cells do not benefit from the indirect anti-tumor effects of MET occurring *in vivo* such as the reduction of insulin levels—where insulin is known to have a mitogenic effect—and cultured cells are exposed to high concentrations of growth factors and glucose present in the culture medium, which may help explain the required higher doses of MET [41].

The anti-tumorigenic properties of MET have been reported in several studies associated with indirect action (reduced insulin levels) or direct actions on molecular pathways that regulate breast tumor cell growth and death [42]. MET may mediate its effects through actions on different cells of the tumor microenvironment, including MOs-macrophages that would be involved in controlling tumor cell growth and progression. However, the activities of immune cells could undoubtedly change when they are in contact with tumor cells. In this context, we investigated the effect of MET on functional activities of autologous MOs cultured alone and when co-cultured with primary breast cancer cells. In conclusion, our results demonstrate a significant effect of MET during MOs-breast cancer cells crosstalk.

Here, we first tested the effects of MET on proliferation and viability of cancer cells. We found that MET downregulated both the proliferation and viability of breast cancer cells. Our results are consistent with those obtained recently using BrdU and 3-(4,5-Dimethylthiazol-2-yl)-2,5-diphenyltetrazolium bromidefor (MTT) assays on breast cancer cell lines MCF-7, MDA-MB-231 and MDA-MB-435 [17,43]. The same effects were observed on MCF-7 and MDA-MB-231 cell viability with the doses of at 1 mM and 5 mM of MET using TBET assays after 24 h and 48 h of treatment [44]. In terms of cell activation and proliferation, the PI3K/Akt/mTOR signaling pathway has been highlighted to play an important role in vital cell functions including cell growth, proliferation, differentiation and survival [45]. Its hyper-activation can lead to excessive tumor cell proliferation, inhibition of apoptosis, angiogenesis, invasion and metastasis [46–48]. For our part, at the concentration of MET used (2.5 mM) we found a modest and non significant reduction of the PI3K/Akt activation in breast cancer cells. However, several studies reported that metformin reduced the survival and proliferation rate of breast cancer cells through the inhibition of PI3K/Akt signaling pathway [49,50].

The phagocytic activity of MOs is the subject of several studies under normal and pathological conditions, including breast cancer [51]. In our study, we observed that pretreatment with MET downregulated the phagocytic capacity of MOs, which is of interest, knowing that high phagocytic capacity is associated with MOs that promote survival and extravasation of cancer cells, and characterizes the so-called ’classical MOs’ in humans [52,53].

Measuring levels of proliferation and phagocytosis in co-culture systems was uninformative because BrdU incorporation and anti-Akt antibodies used for viability and proliferation assays do not discriminate for one cell type or the other in the co-culture [54–56] and the bacteria used for phagocytosis assay have the ability to invade and replicate within several types of phagocytic and nonphagocytic cells, including epithelial cells and this invasion can lead to apoptosis [10,57].

It is well known that necrosis and iNOS are both involved in tissue damage occurring during inflammation. Our results showed that MET treatment had no effect on MOs necrotic death as demonstrated by LDH-based cytotoxicity. However, MET might ameliorate the co-culture effect on necrosis, which is in agreement with previous *in vitro* studies, carried out on BT-20 breast cancer cell line [18]. In MOs, as well as in other cells especially macrophages, the amino acid L-arginine is also used as a substrate by arginase to produce polyamines [58] that contribute to the tumor progression [59], and by iNOS to produce NO [58], which has antitumor effects at high levels [60]. The current study provides evidence that MOs cultured with breast cancer cells exhibited high levels of iNOS, but remain without marked change when treated with MET. Our observations are in agreement with earlier findings demonstrating that MET induces antitumoral activity of macrophages during breast cancer [61] and suppresses polarization toward pro-tumoral phenotypes [62]. Although arginase activity was downregulated in untreated co-cultured cells, it was reversed after MET treatment. Hence, MET has been reported to have opposing effects in normal or pathological conditions whereby it can both attenuate NO production and enhance arginase activation in MOs and macrophages [63,64]. It would be of interest to check the impact of MET treatment on iNOS and arginase activities simultaneously.

The link between cancer and altered metabolism has previously been suggested as a common feature of cancerous tissues, such as the Warburg effect, in which some antioxidant molecules can be used in protective mechanisms against oxidative stress and ROS that are produced during rapid cell proliferation [65]. High levels of ROS can cause macromolecular damage, that can lead to apoptosis and senescence [66]. Our findings demonstrated that catalase activity was downregulated in MET-untreated co-cultures, whereas the co-culture effect on catalase activity can be reversed by MET treatment. Additionally, SOD activity was changed only in MET-treated MOs. In summary, MET treatment did not show metabolic alterations with regard to the levels of the antioxidant molecules catalase and SOD within the co-cultures of MOs with breast cancer cells compared to MOs cultivated alone.

_if_Ca^2+^ is an important secondary messenger that regulates various cellular processes and signaling pathways including those related to cancer, such as apoptosis, proliferation and metastasis [67,68], and those involved in immune responses of MOs, including the production of cytokines and phagocytic activation [69,70]. We first observed that _if_Ca^2+^ levels were reduced in co-culture of MOs with breast cancer cells. In contrast, MET treatment induced _if_Ca^2+^ upregulation in MOs and might reverse the effect of co-culture. So, it has been reported that increased levels of calcium ions are related to both MO activation and the induction of apoptosis in breast cancer cells [68,71,72].

It is now accepted that IL-10 enhances cancer immune surveillance and suppression of cancer-associated inflammation [73], as well as inducing expression of IFN-γ [74], which exerts antitumor activities directly by enhancement of tumor cells antigenicity, inhibition of cell proliferation, the induction of apoptosis or indirectly by inhibition of angiogenesis [75]. Our results indicate that the levels of both IL-10 and IFN-γ decreased during interplay between MOs and breast cancer cells. Except with IL-10, treatment with MET has shown marked differences between its action when MOs are cultured alone and when co-cultured with breast cancer cells. MET induced a decrease in IL-10 levels in MOs and, conversely, an increase in IFN-γ levels. However, MET treatment might ameliorate the co-culture effect on the production of both cytokines, although the differences were not significant for IL-10. So, the results did not show significant differences for IL-10 between cultures of MOs alone, in the absence of treatment, and cultures of MOs with breast cancer cells, treated with MET. These suggest that MET could contribute to the conservation of IL-10 expression levels when MO comes into contact with cancer cells, without inducing changes usually seen after interplay with cancer cells. Our results demonstrate that MET treatment is clearly effective and important in a system that shares some similarities with the biological system where mononuclear cells are not alone but may be confronted with malignant cells.

Of note, circulating MOs can exert different roles based on phenotype characterization. Hence, it has been reported that circulating monocytes subsets so-called classical inflammatory monocytes (moM1, CD14^++^/CD16^-^) and patrolling non-classical monocytes (moM3, CD14^+^/CD16^++^) exert opposite effects after their recruitment in tumor microenvironment *via* the chemokine (C-C motif) ligand 2 (CCL2), also referred to as monocyte chemoattractant protein 1 (MCP1) and small inducible cytokine A2, which is secreted by malignant cells. moM1 can be recruited to the tumor microenvironment where they can be transformed into tumor-associated macrophages (TAMs) that facilitate tumorigenesis by suppression of CD8^+^ T-cell function, recruitment of regulatory T (Treg) cells, angiogenesis, tumor cell intravasation, and metastasis [76], while moM3 display an anti-tumoral role, by directly engulfing cancer cells and by releasing CCL3, CCL4 and CCL5 chemokines, which in turn induce recruitment and activation of cytotoxic natural killer (NK) cells [52]. Another subset of circulating and tumor-associated monocytes endowed with proangiogenic activity, are characterized by the expression of the TIE-2/Tek angiopoietin receptor, Endoglin and VEGF-R2 in the intermediate monocytes subset (moM2, CD14^++^/CD16^+^) as confirmed by a genomic analysis [77]. TIE-2 expressing monocytes (TEM) are recruited to the tumor site where they have been shown to be essential for angiogenesis and inhibition of tumor cell apoptosis by mechanisms depending on TNF-α release. They also have the highest capacity to induce CD4^+^ T-cell activation [78]. Therefore, it would be of interest to investigate MET effects on all characterized MO subsets before and after their individual crosstalk with the studied autologous breast cancer cells. The specific objective being to see whether or not MET treatment would reverse the pro-tumoral activities of moM1 and moM2, knowing that monocytes are endowed with plasticity and versatility in their phenotype [79,80].

## Conclusions and future prospects

*In fine*, our results not only show that the activities of human MOs change when they interact with autologous primary breast cancer cells, but also provide the first evidence that MET treatment can have a potent role in reversing the effects of the crosstalk between MOs and breast cancer cells, especially on the production of co-operative ‘antitumor IFN-γ’ and ‘regulatory IL-10’ cytokines, intracellular calcium signals, as well as immune-metabolic and protective redox based-biomarkers as summarized in the graphical abstract (S2 Fig). These findings open the route to further investigations including the study of MET on autophagy/reverse Warburg effects, as well as the molecular characterization of different subsets of monocytes involved in the interaction with breast cancer cells following MET treatment.

## Supporting information

S1 Fig (DOCX)

S2 Fig (DOCX)

S3 Fig (PDF)
