# Supplementary material for: Metformin partially reverses the inhibitory effect of co-culture with ER-/PR-/HER2+ breast cancer cells on biomarkers of monocyte antitumor activity
Source: PLoS One. 2020 Oct 27;15(10):e0240982. doi: 10.1371/journal.pone.0240982 (PMC7591052; doi:10.1371/journal.pone.0240982)
Supplement: S1 Fig — (DOCX) [file pone.0240982.s001.docx]

**
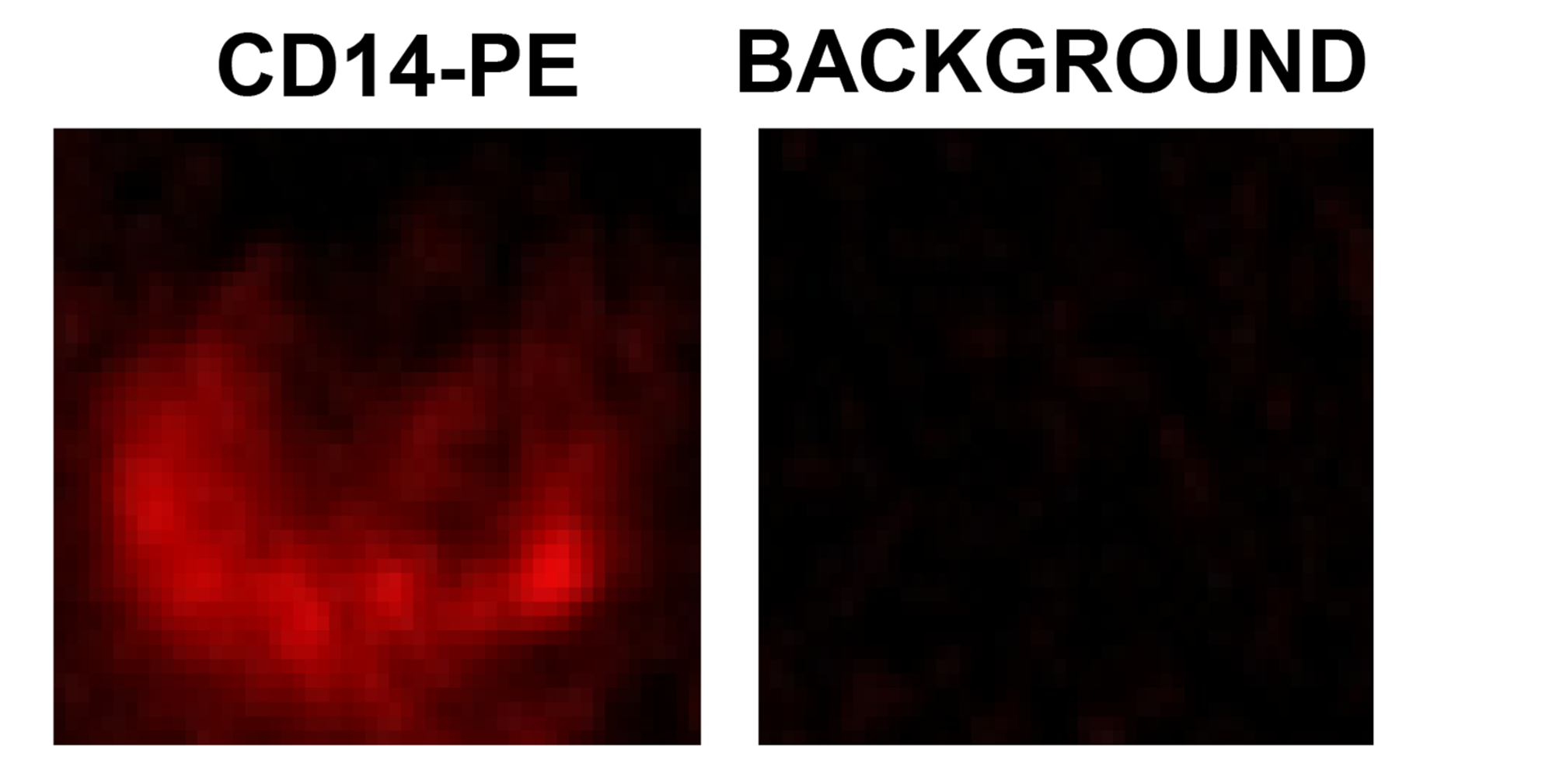
**

**S1 Fig. Supplementary methods.**

**Purity of MOs isolated from PBMCs**

The purity of MOs was verified by direct immunofluorescence using inverted cell imaging fluorescence microscopy station (Floid Cell Imaging Station, Thermo Fischer Scientific, MA USA). 2 × 10^5^ of MOs were cultured at 37 °C and 5% of CO_2_ in chamber slide. After fixation by 4% paraformaldehyde (PFA) for 15 min and blocking by 5% FBS in PBS for 1 h, MOs were stained with PE-conjugated antihuman CD14 (BD Biosciences, San Diego, CA, USA) diluted at 1:750 in block solution (1). The positive cells were assessed by Floid Cell Imaging Station and counted with ImageJ software (the National Institutes of Health, Bethesda, MD, USA) (2,3). The purity of MOs was around 90%.

**References**

1. Belhassena I, Nouari W, Messaoud A, Nouar M, Brahimi M, Lamara S-AC, Aribi M. Aspirin enhances regulatory functional activities of monocytes and downregulates CD16 and CD40 expression in myocardial infarction autoinflammatory disease. *Int Immunopharmacol* (2020) **83**:106349. doi:10.1016/j.intimp.2020.106349

2. Jackson M, Krasnodembskaya A. Analysis of Mitochondrial Transfer in Direct Co-cultures of Human Monocyte-derived Macrophages (MDM) and Mesenchymal Stem Cells (MSC). *BIO-Protoc* (2017) **7**: doi:10.21769/BioProtoc.2255

3. Schneider CA, Rasband WS, Eliceiri KW. NIH Image to ImageJ: 25 years of image analysis. *Nat Methods* (2012) **9**:671–675. doi:10.1038/nmeth.2089
