# Supplementary material for: Metformin partially reverses the inhibitory effect of co-culture with ER-/PR-/HER2+ breast cancer cells on biomarkers of monocyte antitumor activity
Source: PLoS One. 2020 Oct 27;15(10):e0240982. doi: 10.1371/journal.pone.0240982 (PMC7591052; doi:10.1371/journal.pone.0240982)
Supplement: S2 Fig — (DOCX) [file pone.0240982.s002.docx]

**
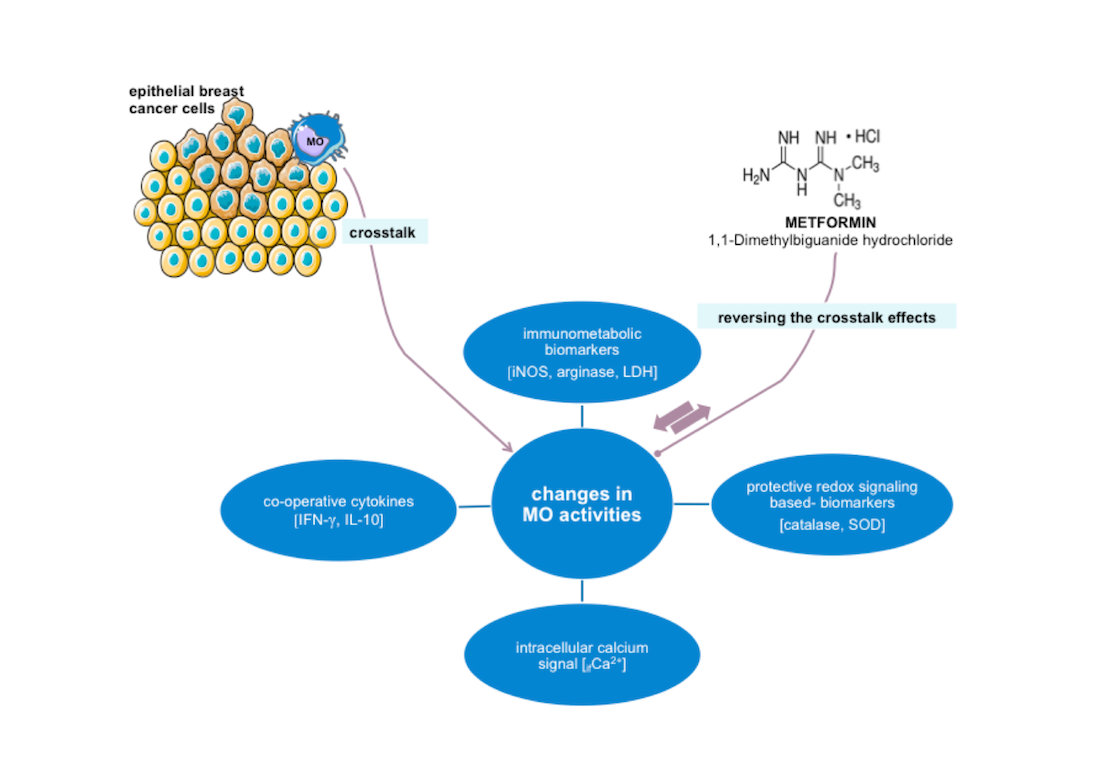
**

**S2 Fig. Graphical Abstract**

Dahmani et al. have shown that the interaction of human monocytes with autologous primary breast cancer cells (ER^-^/PR^-^/HER2^+^) induces a change in their functional phenotypic activities, affecting metabolic biomarkers (inducible nitric oxide synthase [iNOS] activity, arginase activity, LDH-based cytotoxicity), protective redox signaling based-biomarkers (catalase activity, superoxide dismutase [SOD] activity), intracellular free calcium ions (_if_Ca^2+^), and the production of co-operative ‘antitumor/immunostimulatory IFN-γ’ and ‘immunoregulatory/immunosuppressive IL-10’ cytokines, as demonstrated by co-culture systems. Of great interest, the study provides the first evidence that metformine treatment can have a potent role in reversing these effects.
