# Supplementary figures and images for: Metformin partially reverses the inhibitory effect of co-culture with ER-/PR-/HER2+ breast cancer cells on biomarkers of monocyte antitumor activity
Source: PLoS One. 2020 Oct 27;15(10):e0240982. doi: 10.1371/journal.pone.0240982 (PMC7591052; doi:10.1371/journal.pone.0240982)

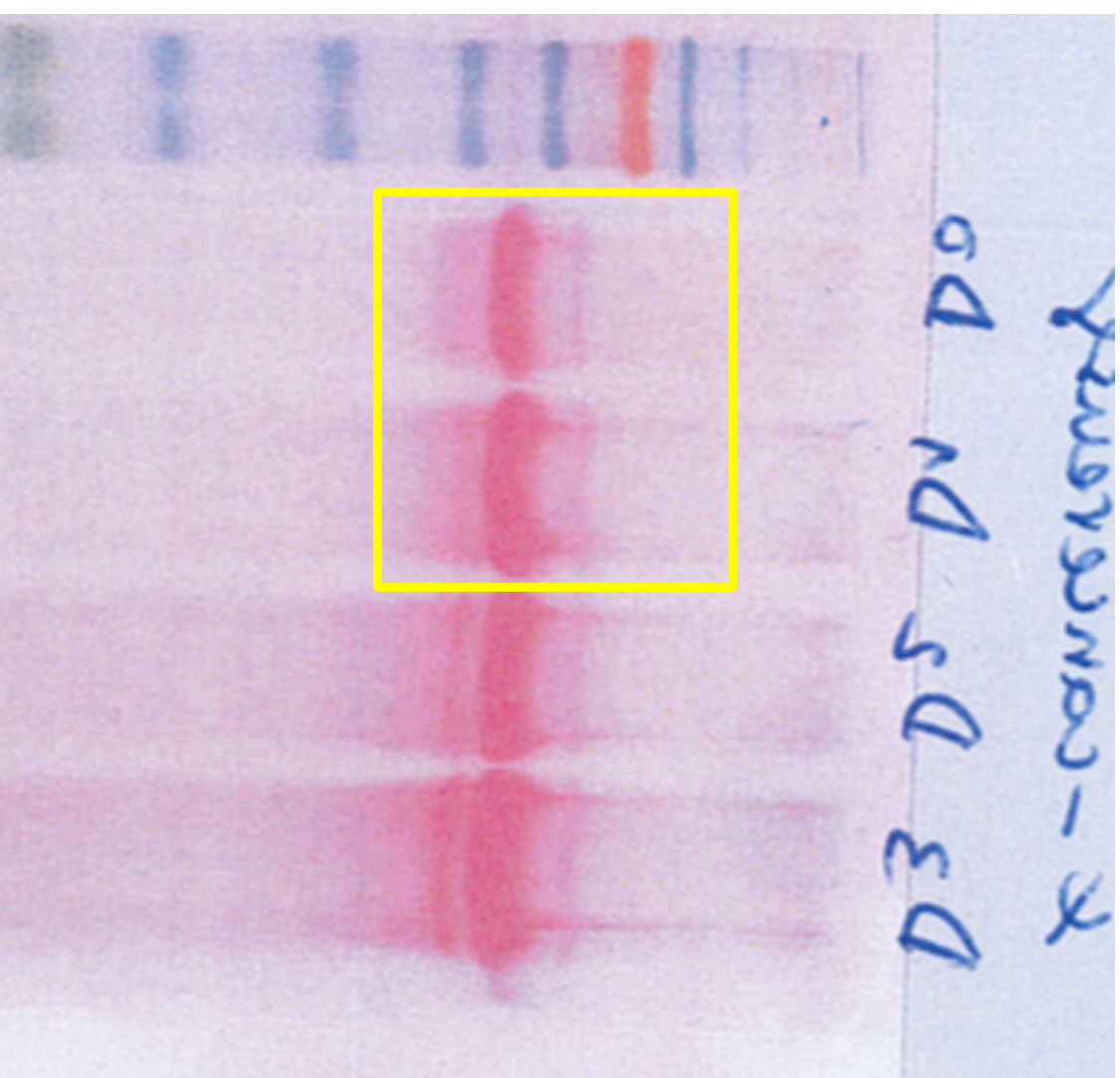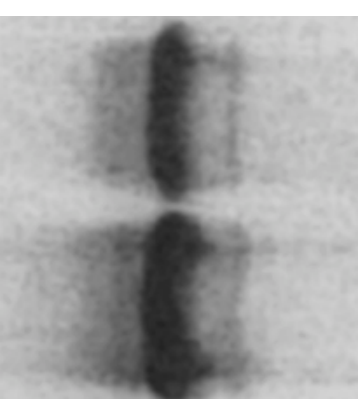

Ponceau Red

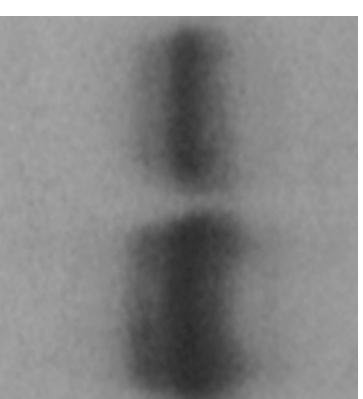

Phospho-Akt

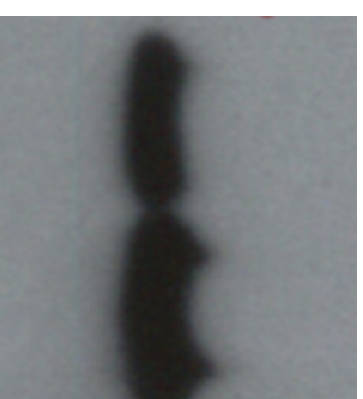

Anti-Akt2

Supplement: S3 Fig — (PDF) [file pone.0240982.s003.pdf]
